# Controlling the transverse proton relaxivity of magnetic graphene oxide

**DOI:** 10.1038/s41598-019-42093-1

**Published:** 2019-04-04

**Authors:** Bibek Thapa, Daysi Diaz-Diestra, Dayra Badillo-Diaz, Rohit Kumar Sharma, Kiran Dasari, Shalini Kumari, Mikel B. Holcomb, Juan Beltran-Huarac, Brad R. Weiner, Gerardo Morell

**Affiliations:** 1 Molecular Sciences Research Center, University of Puerto Rico, San Juan, PR 00926 USA; 2 Department of Physics, University of Puerto Rico, Río Piedras Campus, San Juan, PR 00925 USA; 3 Department of Chemistry, University of Puerto Rico, Río Piedras Campus, San Juan, PR 00925 USA; 4 Department of Biology, University of Puerto Rico, Río Piedras Campus, San Juan, PR 00925 USA; 5 Department of Environmental Sciences, University of Puerto Rico, Río Piedras Campus, San Juan, PR 00925 USA; 6 Department of Electrical and Computer Engineering, University of Illinois at Urbana-Champaign, Urbana, IL 61801 USA; 7 Department of Physics & Astronomy, West Virginia University, Morgantown, WV 26506 USA; 8 Center for Nanotechnology in Drug Delivery, UNC Eshelman School of Pharmacy, University of North Carolina, Chapel Hill, NC 27599 USA

## Abstract

The engineering of materials with controlled magnetic properties by means other than a magnetic field is of great interest in nanotechnology. In this study, we report engineered magnetic graphene oxide (MGO) in the nanocomposite form of iron oxide nanoparticles (IO)-graphene oxide (GO) with tunable core magnetism and magnetic resonance transverse relaxivity (r_2_). These tunable properties are obtained by varying the IO content on GO. The MGO series exhibits r_2_ values analogous to those observed in conventional single core and cluster forms of IO in different size regimes—motional averaging regime (MAR), static dephasing regime (SDR), and echo-limiting regime (ELR) or slow motion regime (SMR). The maximum r_2_ of 162 ± 5.703 mM^−1^s^−1^ is attained for MGO with 28 weight percent (wt%) content of IO on GO and hydrodynamic diameter of 414 nm, which is associated with the SDR. These findings demonstrate the clear potential of magnetic graphene oxide for magnetic resonance imaging (MRI) applications.

## Introduction

Magnetic materials such as superparamagnetic iron oxide nanoparticles (IO) have attracted mounting interest for a wide range of applications in nanomedicine^1–4^, magneto-mechanical actuation^5^, energy storage^6–8^, optoelectronics^9,10^, and environmental remediation^11–15^ due to their biocompatibility, hydrophilicity, distinct morphology, and unique magnetic and electric properties. In medical diagnostics, the MRI applications of such materials are of paramount interest, and they are widely used as MRI negative contrast agents (CAs)^16,17^ due to their hallmark characteristics of spin-spin or transverse relaxation enhancement.

In pursuit of high-performance MRI CAs, the surface modification of IO is mostly executed in the form of core-shell^18,19^ and Janus structure^20^ using polymer stabilizers along with their controlled shape and size. In addition, with the advances in the research of graphene-based materials, GO has been utilized for surface modification of IO owing to its oxygenated functionalities, i.e., epoxide, hydroxyl, carbonyl, and carboxyl moieties^21,22^ and biocompatibility^23,24^. These functionalities can serve as the conjugation sites for IO to form GO-based nanocomposites, and in particular, magnetic graphene oxide (MGO). Recently, IO/GO-based nanocomposites have been proposed as T_1_^25^ and T_2_ CAs^4,26,27^ for MRI. However, no systematic studies on the tunable magnetic behavior and magnetic resonance (MR) relaxivity of such materials have been reported, and its corresponding size regime correlation remains unsettled.

Some groups have recently explored the tunability of magnetic resonance transverse relaxivity (r_2_) in single core or cluster forms of IO in the PEGylated core-shell nanostructures. The distinct r_2_ values of IO with size range ~5–14 nm were reported via optimization of the coating thickness using PEG with molecular weights of 550, 750, 1000, 2000 and 5000 Da^28^. Similarly, the PEGylated raspberry-like nanoclusters of superparamagnetic IO nanocrystals with a diameter range of 30 to 200 nm were reported with distinct r_2_ values associated with three size regimes–motional averaging regime (MAR), static dephasing regime (SDR), and echo-limiting regime (ELR) or slow motion regime (SMR)^29^, as defined by outer sphere relaxation theory^30,31^.

In this study, we synthesize a series of MGO with different concentration of IO and demonstrate that these MGOs possess such size regimes with distinct r_2_ values. We assume that the MGO behaves as a spherical system with a size characterized by its hydrodynamic diameter which can be tuned by changing the concentration of the IO, i.e., the higher the concentration of IO, the larger is the size of MGO, and that the MGO can translationally diffuse water molecules on its outer sphere region, makes them experience diverse magnetic field gradients induced by it, and in turn lead to enhanced r_2_. Based upon this assumption, we present a detailed study on the control of r_2_ of MGOs as a function of concentration of IO. Further, we aim at maximizing the transverse relaxivity through different MGO size regimes and determining its correlation to that observed in conventional single core and cluster forms of IO. More importantly, we developed an intriguing strategy for the synthesis of magnetic graphene oxide capable of governing the tunable transverse relaxivity, which used to be elusive on such nanocomposite materials.

## Results and Discussion

**Figure 1:**
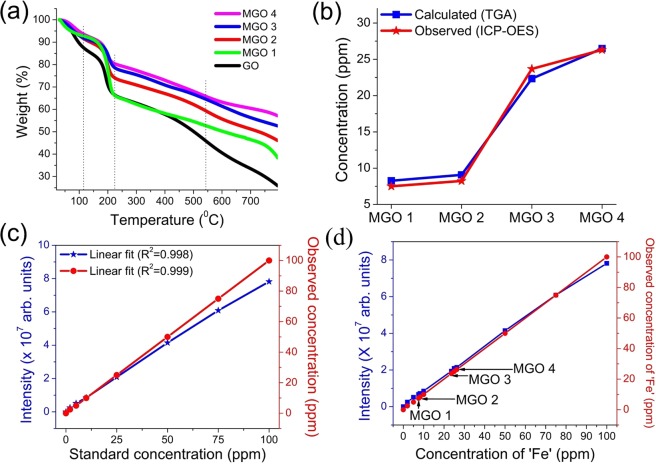
(**a**) Thermogravimetric analysis (TGA) profile. (**b**) A plot of calculated iron (‘Fe’) concentrations from TGA and observed from inductively coupled plasma optical emission spectrometry (ICP-OES). (**c**) The calibration linear fit of the standard solutions from ICP-OES. (**d**) Intensity and concentration plot obtained from ICP-OES.

We have synthesized MGO samples (MGOs), namely MGO 1, MGO 2, MGO 3 and MGO 4, with 8, 18, 28 and 32% (w/w) of IO on GO respectively in nanocomposite form (see the Methods section for details). Firstly, we employed thermogravimetric analysis (TGA) for the quantification of IO percentages on GO using 7 mg of each sample. Fig. 1a illustrates the TGA profile for GO, MGO 1, MGO 2, MGO 3 and MGO 4. For all MGO samples, ~7% weight loss was seen in the initial stage below 120 °C which was due to the evaporation of the water molecules adsorbed by GO, whose surface is hydrophilic^32^. However, GO showed the prompt weight loss of 13% at this temperature due to the absence of IO. In the next stage, the weight loss was more rapid below 226 °C which is attributed to the pyrolysis and thermal decomposition of oxygen functionalities including −OH, −COOH and −COOR, to produce H_2_O, CO, and CO_2_^33^. In this stage, the presence of diverse percentages of IO on GO in different samples can be perceived with the distinct split of TGA curves. The respective mass losses for the MGOs are noted to be 35%, 26%, 22%, and 20%. The weight loss between 450 °C to 600 °C was due to combustion of the carbon skeleton. 25% of the carbon content was left after heating to 800 °C, and the magnetite (Fe_3_O_4_) was completely oxidized to hematite (Fe_2_O_3_) at this temperature^34^. Based on these observations, the respective IO content in the MGOs was determined to be 10%, 20%, 27% and 32% (w/w) respectively. Using the TGA technique, the iron oxide content in the iron oxide-graphene composite^35^ and iron oxide-nitrogen doped reduced graphene oxide was determined previously and was consistent with those calculated from the synthesis.

The ICP-OES technique was used to determine the ‘Fe’ concentrations in MGOs for the confirmation of IO content in the MGOs. The data of calculated ‘Fe’ concentrations based upon the wt% of IO observed from TGA, and the experimentally observed ‘Fe’ concentrations from ICP-OES are tabulated in Table S1 in the supporting information (SI). As shown in Fig. 1b,d, both the calculated and observed ‘Fe’ concentrations are in reasonable agreement; however, the calculated ‘Fe’ concentrations in MGO 1 and MGO 2 are marginally higher than that observed from ICP-OES, while it is lower for MGO 3. Based upon these results, we further averaged the values of IO wt% to 8% IO@GO in MGO 1, 18% IO@GO in MGO 2, 28% IO@GO in MGO 3 and 32% IO@GO in MGO 4. The ‘Fe’ wt% was also quantified by energy-dispersive X-ray (EDX) spectroscopy of MGOs, and the analysis was done on three different spots of each MGO (Fig. S1 in SI). It was observed that the MGOs show increasing wt% of IO from MGO 1 to MGO 4 which is consistent with the TGA and ICP-OES measurements.

**Figure 2:**
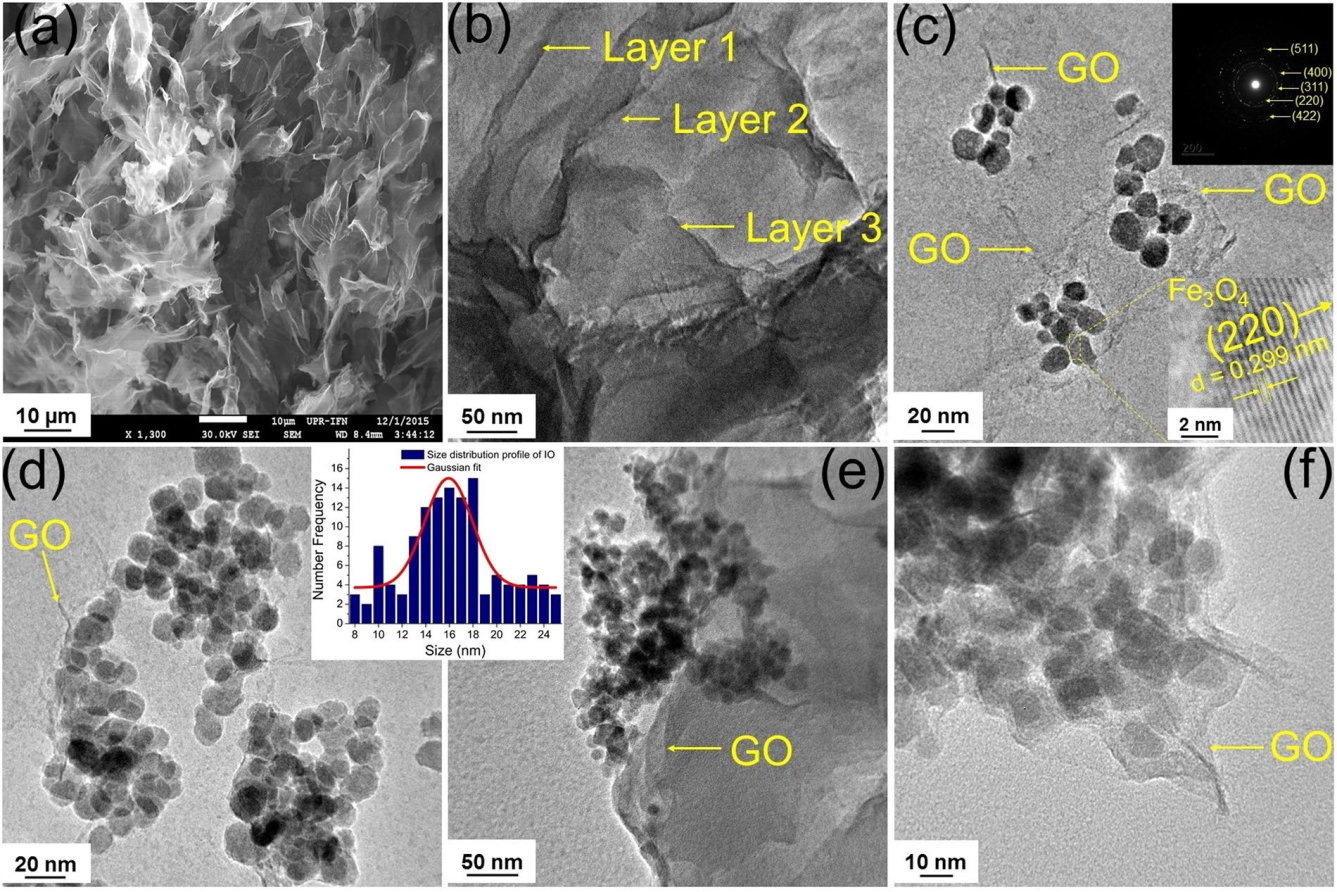
FE-SEM image of (**a**) as-synthesized GO. TEM images of (**b**) as-synthesized GO; (**c**) MGO 1 (8% IO@GO), inset top right: selected area electron diffraction (SAED) pattern of IO, inset bottom right: HR-TEM image showing lattice fringes of (220) plane of magnetite (Fe_3_O_4_) phase; (**d**) MGO 2 (18% IO@GO); (**e**) MGO 3 (28% IO@GO); and (**f**) MGO 4 (32% IO@GO). Inset of Fig. 2d,e corresponds to the size distribution profile of IO particles alone.

The morphology and nanostructure of the synthesized GO and MGOs were observed using field emission scanning electron microscopy (FE-SEM) and field emission transmission electron microscopy (FE-TEM). The aggregated but well-exfoliated GO flakes are clearly seen in Fig. 2a,b obtained from FE-SEM and FE-TEM respectively. The agglomerated quasi-spherical IO, which is the hallmark of the co-precipitation synthesis^36^, decorated on GO are seen in MGO 1, MGO 2, MGO 3 and MGO 4, as depicted in Fig. 2c–f respectively. The inset in Fig. 2c shows the high resolution TEM (HR-TEM) image of the IO and shows the polycrystalline nature with an interplanar lattice spacing of 0.299 nm corresponding to the characteristic spacing of (220) planes of magnetite (Fe_3_O_4_)^36,37^. The IO particle size distribution profile (inset in Fig. 2d,e) shows their size ranging from 8 to 25 nm, but mostly 14 to 18 nm in size, suggesting the formation of polydisperse nanoparticles. The size was determined using ImageJ software (https://imagej.nih.gov/ij/).

**Figure 3:**
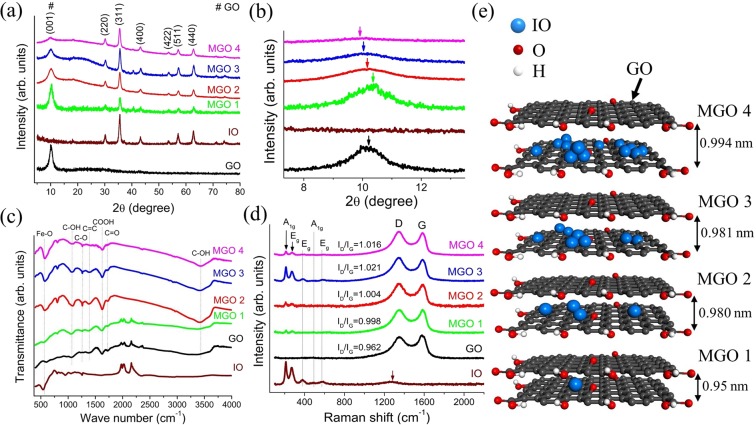
(**a**) XRD patterns of GO, IO, and MGOs. (**b**) XRD peaks shift corresponding to GO observed from (**a**). (**c**) Attenuated total reflectance Fourier transform infrared spectroscopy (ATR-FTIR) spectra of GO, IO, and MGOs. (**d**) Raman spectra of IO, GO and MGOs. (**e**) The schematic representation of IO aggregation in various flakes of GO in MGOs.

The crystallinity and phase formation were analyzed using X-ray diffraction (XRD) as shown in Fig. 3a. The diffraction patterns show the major characteristic peaks corresponding to the magnetite (Fe_3_O_4_) phase and the well-defined peaks indicate the highly crystalline nature of IO^36,37^. The intensity of the diffraction peaks corresponding to GO gradually decreases as the content of IO increases in MGOs suggesting an increased degree of exfoliation of GO (Fig. 3b). The shift of the GO diffraction peak towards lower 2θ values, which is accompanied by decreasing intensity, is consistent with the anticipated increase of the GO interlayer stacking distance in MGOs^4^. The diffraction peak positions observed at 10.14°, 10.34°, 10.11°, 10.02° and 9.93°, correspond to the interlayer stacking distance (d) of 0.970 nm, 0.95 nm, 0.980 nm, 0.981 nm and 0.994 nm, for GO, MGO 1, MGO 2, MGO 3 and MGO 4, respectively (Fig. 3e).

The characteristic transmittance peaks associated with the major functional groups described in TGA analysis were identified in ATR-FTIR spectra. According to the ATR-FTIR spectra (Fig. 3c), the corresponding vibrations at the characteristics peaks positions are: 570 cm^−1^ (Fe–O vibration), 803 cm^−1^ (C–O epoxy stretching vibration), 1069 cm^−1^ (C–O alkoxy stretching vibration), 1261 cm^−1^ (C–O epoxy stretching vibration), 1381 cm^−1^ (O–H bending vibration), 2851 cm^−1^ (CH_2_ asymmetric vibration), 2923 cm^−1^ (CH_2_ symmetric vibration), and 3200 cm^−1^−3700 cm^−1^ (O–H stretching vibration and H_2_O molecules). The Fe–O vibrational mode in MGOs experiences a slight blueshift than in IO, which may be ascribed to its partial confinement between GO layers.

The composition and structural change of IO, GO, and MGOs were examined using Raman spectroscopy. The Raman spectrum of bare IO (see Fig. 3d) shows the characteristic peaks of magnetite (Fe_3_O_4_), in agreement with XRD data. However, the spectrum also shows peaks characteristic of maghemite (Fe_2_O_3_) due to the phase change induced by the focused laser irradiation^38^ in the Raman microprobe employed for the experiment. The two A_1g_ vibrational modes observed at 212 cm^−1^ and 494 cm^−1^ and two E_g_ vibrational modes observed at 270 cm^−1^ and 590 cm^−1^ are associated with the maghemite (Fe_2_O_3_) phase. An E_g_ vibrational mode seen at 378 cm^−1^ represents the characteristic magnetite (Fe_3_O_4_) phase, which is predominant. A noticeable broad band centered at 1280 cm^−1^ (marked by an arrow) is ascribed to the scattering of two magnons resulting from their interaction created on neighboring antiparallel spin sites^39^. The Raman spectra of GO and MGOs show the prevailing peaks corresponding to G and D bands of GO. The G band peak, centered at 1588 cm^−1^, originates from the first order scattering of the E_2g_ mode at Γ-point or the in plane stretching motion between sp^2^ carbon systems. The presence of the D band peak at 1355 cm^−1^ is due to the second-order double resonant process between non-equivalent K points in the Brillouin zone of graphene^40,41^. The D-mode arises by disorder due to the edge, structural defects, asymmetric sp^2^-hybridized carbon systems, and hence the D band is known as a disorder band^42^. The presence of IO induces a structural defect to the basal plane of GO resulting in an intensified D band, and the disorder level in GO can be estimated by the relative intensity of the D and G bands (I_D_/I_G_). The estimated I_D_/I_G_ value (0.962, 0.998, 1.004, 1.021 and 1.016 for GO, MGO 1, MGO 2, MGO 3 and MGO 4, respectively) increases with the IO content in MGOs, which is consistent with the TGA analysis. The prevailing peaks of GO over IO in MGOs are evident of higher GO content in the composite systems.

**Figure 4:**
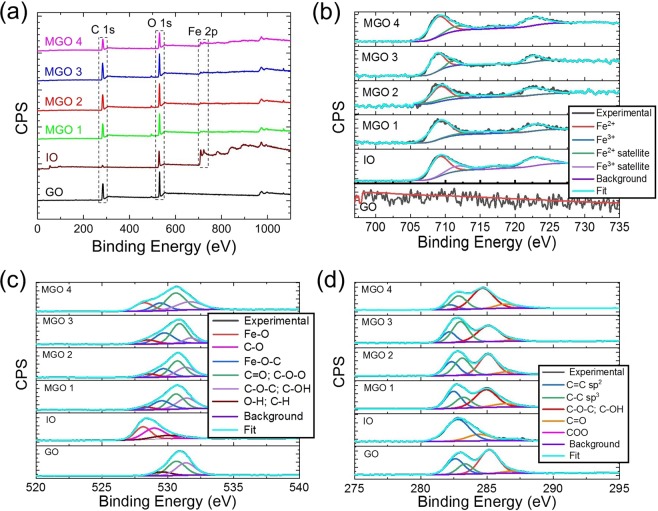
(**a**) XPS survey spectra of GO, IO, and MGOs. High resolution (**b**) Fe 2p spectra, (**c**) O 1s and (**d**) C 1s spectra of GO, IO, and MGOs with the experimental and deconvolution fittings.

X-ray photoelectron spectroscopy (XPS) was employed to study the interactions between IO and GO in MGOs and determine their chemical composition and phases. As observed in Fig. 4a, the survey spectra depict prominent peaks at ~284 and 531 eV associated with C 1s and O 1s in GO, which also co-exist in the spectra of IO and MGOs together with Fe 2p peaks between 709 and 724 eV. The Fe 2p high resolution spectrum of IO (Fig. 4b) shows the binding energy peaks corresponding to the Fe 2p_3/2_ and Fe 2p_1/2_ spin-orbit peaks of Fe_3_O_4_ at 709.5 and 723.1 eV, respectively. This suggests the formation of a mixed oxide of Fe (II) and Fe (III)^43^. These peaks are also clearly seen in Fe 2p high resolution spectra of MGOs indicating the successful formation of IO and GO nanocomposites. The O 1s spectrum of GO (Fig. 4c) depicts three peaks at 529.3, 530.8 and 531.7 eV associated with the oxygen in hydroxyl (O–H; C–H), carboxylate and/or carbonyl (C–O–O; C=O) and epoxy and/or hydroxyl (C–O–C; C–OH), respectively. The peaks of O 1 s in the spectra of MGOs broaden and shift to the lower binding energy towards the characteristics O 1 s of lattice oxygen in Fe_3_O_4_ (528.1 eV; Fe–O)^44^. The formation of a new peak at 529.8 eV in MGOs suggests the binding of IO on GO with the oxygen functionalities via Fe–O–C bond. Moreover, the decrease in the XPS intensities of C–O–C and C–OH in MGOs versus GO indicates the bidentate complex formation between carboxylate group and Fe element^45^.

The high-resolution C 1s spectra of GO in Fig. 4d show the high-intensity peak of C–O–C; C–OH (285.3 eV) compared to that of C=C (sp^2^) (282.6 eV), C–C (sp^3^) (283.5 eV), C=O (287.1 eV) and O–C=O (287.5 eV) indicating that the GO is oxidized by the hydroxyl and epoxy groups. It is evidently seen that the C=C (sp^2^) and C–C (sp^3^) peaks in the MGOs shift to lower binding energy from GO. These binding energy values of C=C (sp^2^)/C–C (sp^3^) expressed as 282.4/283.2 eV, 282.3/283 eV, 282.1/282.7 eV and 282/282.3 eV in MGO 1, MGO 2, MGO 3, and MGO 4, respectively, were shifted indicating the contribution of different loading percentages of IO on GO. However, no significant decrease in the intensities of the peaks from the oxygen functionalities was observed in MGOs.

**Figure 5:**
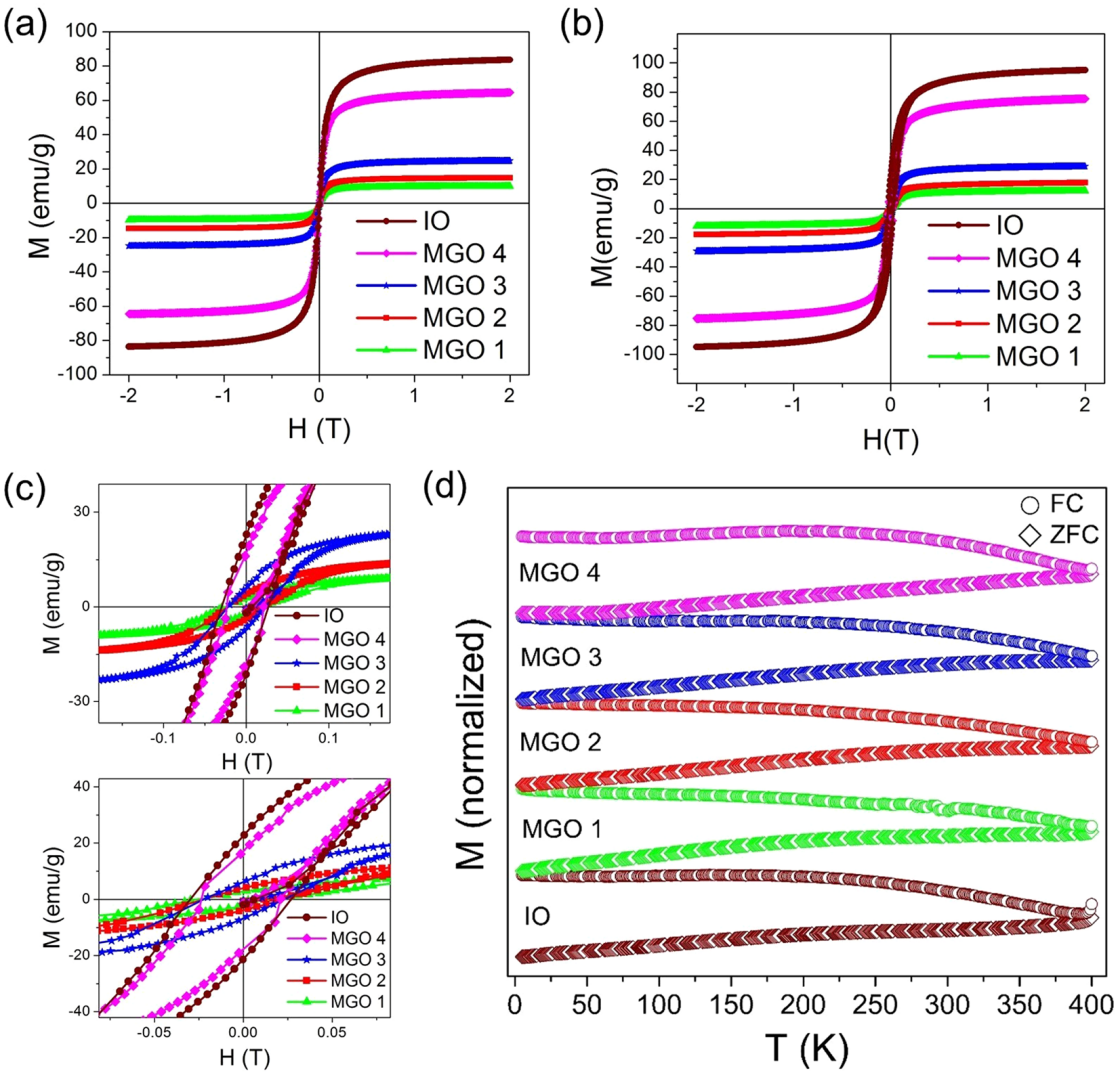
Magnetic hysteresis (*M–H*) curves of IO and MGOs measured at (**a**) 300 K and (**b**) 5 K (**c**) *M-H* curves in low magnetic field region showing negligible coercivity (d) FC and ZFC curves of IO and MGOs.

Magnetic field and temperature dependent magnetization measurements of IO and MGOs were performed in a physical property measurement system (PPMS) using vibrating sample magnetometry (VSM). The field dependent magnetization (*M–H*) was performed at 5 K and 300 K in the range ± 2 T of applied field (Fig. 5a) while the temperature dependent magnetization (*M–T*), field cooled (FC) and zero field cooled (ZFC) magnetic measurements as a function of temperature, were recorded under an external dc field of 5 mT in the temperature range of 5–400 K (Fig. 5b). The IO exhibited a large saturation magnetization (M_S_) of 84 emu/g of ‘Fe’ which increases to 95 emu/g when the temperature is decreased to 5 K, suggesting that the magnetic spins are more oriented along the applied field at low thermal energy (Fig. 5a,b). The M_S_ values of MGOs are observed to be 65, 25, 15 and 10 emu/g for MGO 4, MGO 3, MGO 2, and MGO 1, respectively, at 300 K which increased to 76, 30, 18 and 13 emu/g at 5 K. These values are consistent with the weight percentages of IO in respective MGOs. The increased amount of IO in GO creates higher defects that lead to interaction between the local moments^46^ resulting in higher saturation magnetization in the MGOs. It is reported that GO shows paramagnetic and antiferromagnetic behaviors at low and room temperature, respectively^46^. The lower M_S_ values of MGOs when compared to IO at both 5 K and 300 K are ascribed to the paramagnetic and antiferromagnetic contribution from GO. The magnetic hysteresis (*M–H*) curves of IO and MGOs measured at 300, and 5 K exhibit typical superparamagnetic behavior with negligible coercivity (H_C_) (Fig. 5c, top, and bottom)

The M_ZFC_ (T) curves of all MGOs and IO clearly show irreversibility over a wide temperature range as shown in Fig. 5d. This behavior indicates that the thermal energy of the magnetic spins remains insufficient to overcome the anisotropy energy barrier of the IO clusters up to 400 K and therefore, remain blocked. From the M_ZFC_ (T) curves, it is observed that a 50 mT static magnetic field is not sufficient for rapid alignment of the magnetic moments. A gradual increase in the magnetic moment without forming well-defined blocking temperature (T_B_) is observed as the temperature is raised. The observed behaviors are analogous to those reported in iron-carbon nanocomposites^47^. The broad M_ZFC_(T) curves suggest the presence of a distribution of blocking temperatures due to the distribution of energy barriers governed by the size variation of IO. A plateau-shape in the M_FC_ (T) curve of MGO 4 below 55 K is associated with the freezing of the magnetic moments of iron oxide nanoparticles, resulting in spin-glasslike behavior.

**Figure 6:**
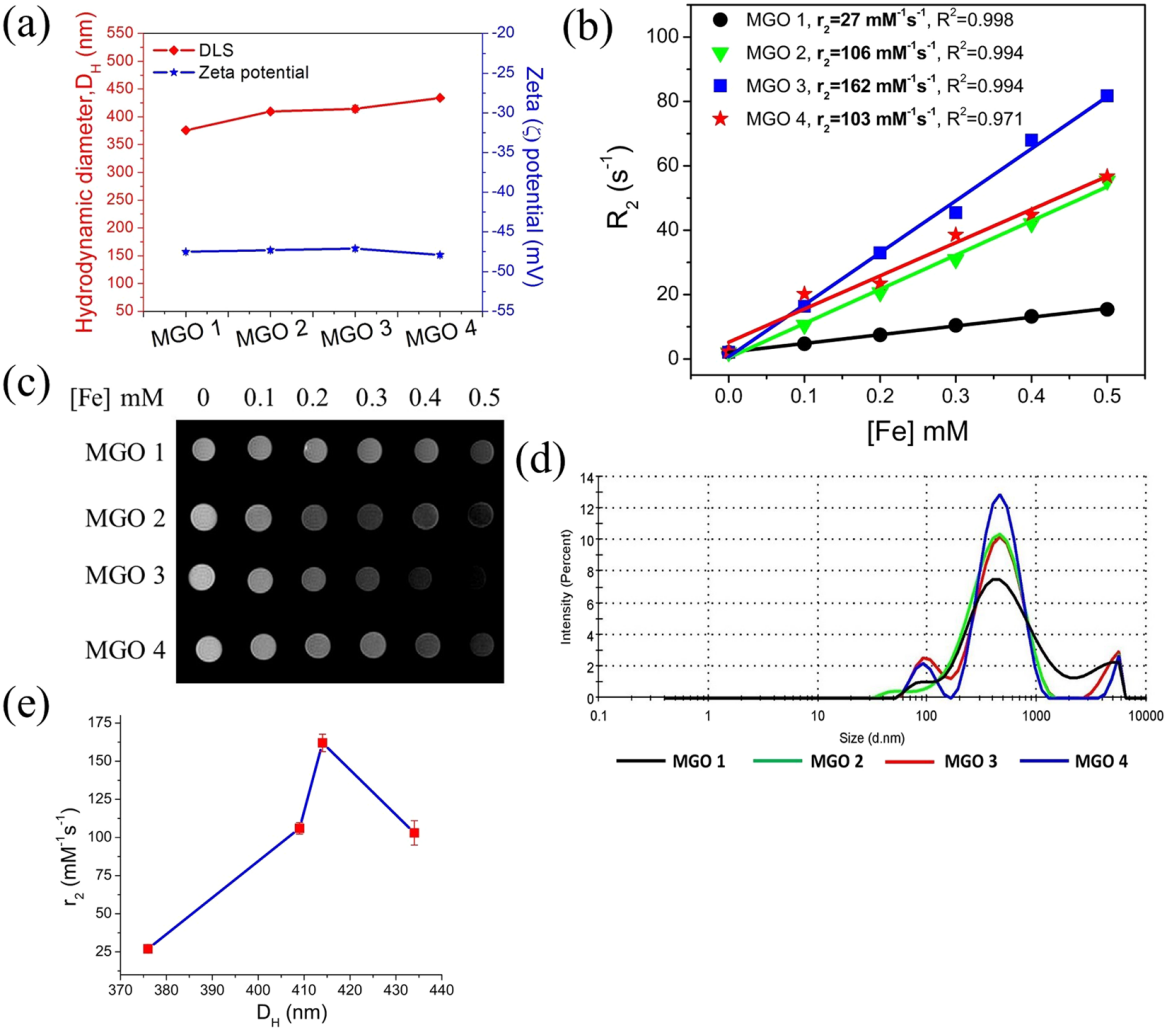
(**a**) Dynamic light scattering (DLS) and Zeta potential, (**b**) MR transverse relaxivity (r_2_) measurements, (**c**) MR *in vitro* phantom images, (**d**) DLS size (D_H_) distribution profile of MGOs and (**e**) r_2_ values with respect to D_H_ of MGOs.

The aqueous dispersibility of such materials is one of the main properties in the design for the MRI applications. The dynamic light scattering (DLS) and Zeta potential measurements were carried out for the investigation of hydrodynamic size (D_H_) and the aqueous stability of MGOs. The D_H_ for MGO 1, MGO 2, MGO 3, and MGO 4 are recorded to be 376 nm, 409 nm, 414 nm, and 434 nm, respectively, which is much larger than that of the IO particles alone (8–25 nm as shown in Fig. 2), as expected. The observed D_H_ (Fig. 6a) for MGOs are consistent with the IO content in GO. The MGOs demonstrate excellent aqueous dispersibility with a Zeta potential in the range of −47 to −48 mV, essentially constant, which suggests the presence of abundant oxygenated functionalities in GO^48^. The schematic representation and optical images of the aqueous dispersed MGOs and their magnetic capture are shown in Fig. S2 in SI.

The measurements of transverse relaxivity (r_2_) of MGOs were performed at 1.41 T. The relaxivity values were calculated from the slope of the relaxation rate (R_2_ = 1/T_2_) versus concentration of iron ([Fe]) graph shown in Fig. 6b, where T_2_ is the protons’ transverse relaxation time at a given concentration of iron. The MGO 1 exhibits the lowest r_2_ of 27 ± 0.53 mM^−1^s^−1^ (R^2^ = 0.998) while r_2_ increases to 106 ± 3.75 mM^−1^s^−1^ (R^2^ = 0.994) in MGO 2. Similarly, the MGO 3 exhibits the highest r_2_ value at 162 ± 5.70 mM^−1^s^−1^ (R^2^ = 0.994) with 28% of IO and r_2_ decreases to 103 ± 8.00 mM^−1^s^−1^ (R^2^ = 0.971) in MGO 4 as the IO content increases to 32%. The exponential decay curves (Fig. S3 in SI) show the steady decrease in transverse intensity with the increase in [Fe]. The size distribution profile of MGOs is shown in Fig. 6d to correlate with the dependency of r_2_ on the D_H_ of MGOs (Fig. 6e) and point to a close analogy to the r_2_ values associated with the theoretical size regimes, MAR, SDR, and SMR, which are specified in classical outer sphere relaxation theory.

The dependence of r_2_ on the size of IO is directly related to how far the water molecules (protons) diffuse with respect to the size of IO, *i.e*., the diffusion length of water protons relative to the size of IO, as given by the relation^30^,

1 $${{\rm{\tau }}}_{{\rm{diff}}}={{\rm{D}}}^{2}/4\,{{\rm{C}}}_{{\rm{diff}}}$$

where τ_diff_ is the diffusion time of water protons, D is the diameter of IO and C_diff_ is the translation diffusivity of water protons. The r_2_ values for MGO 1 and MGO 2, with D_H_ of 376 nm and 409 nm, respectively, indicate that the MAR condition was achieved signifying that the randomly diffusing water protons experience diverse susceptibility gradients with increasing D_H_, which are time-averaged and r_2_ is given by the relation^31^,

2 $${{\rm{r}}}_{2}=(16/45)\,{\rm{\gamma }}\,{{\rm{\tau }}}_{{\rm{D}}}{({\rm{\Delta }}{\rm{\omega }})}^{2}$$

for τ_diff_ (Δω) < 1, where γ is the volume fraction of IO, Δω = γ(B_eqtr_) = γ_0_(μ_0_M_S_/3) is the change in Larmor frequency of protons at the equator of MGO that generates the magnetic field of B_eqtr_, M_S_ is the saturation magnetization of MGO, γ_0_ = 2.67 × 10^8^ rad·s^−1^·T^−1^ is the gyromagnetic ratio of protons, and μ_0_ = 4π·10^−7^ T·m·A^−1^ is the magnetic permeability in vacuum.

Similarly, MGO 3 attains the maximum r_2_ value with D_H_ = 414 nm corresponding to the SDR condition, i.e., the distance traveled by diffusion is less than the characteristic separation of IO due to the higher D_H_ value, and the water protons do not experience a significant susceptibility gradient, thus yielding maximum r_2_. The SDR is achieved when τ_diff_ (Δω) > 1 and r_2_ is given as^31^

3 $${{\rm{r}}}_{2}=(2\pi /9)\,\{{\gamma \gamma }_{0}({\mu }_{0}{{\rm{M}}}_{{\rm{S}}})\}$$

Further increasing the D_H_ of IO beyond the SDR condition, the increase in r_2_ ceases by echo-limiting or T_2_*-limiting and r_2_ decreases consequently, and this size regime corresponds to the ELR or SMR condition. This depends on the magnitude of diffusion time (τ_D_$$)$$ and the echo time (τ_TE_), i.e., the time between 90° RF pulse and the peak of echo signal induced. The refocusing 180° RF pulse being antiparallel to the initial 90° RF pulse inverts the dephasing and refocuses the protons to in phase. This slows down the decay of transverse intensity to be slower than in the SDR condition and r_2_ decreases, which was observed in MGO 4 with D_H_ of 434 nm.

Also, we carried out MR *in vitro* T_2_-weighted MR phantom imaging on MGOs to assess their T_2_ contrast enhancement efficacy. The imaging was performed in DI water at 4.7 T using multiple spin-echo sequences with a repetition time (TR) = 12000 ms, echo time (TE) = 24 ms, field of view (FOV) = 60, phase = 40 and thickness = 1.50 mm. The phantom images, in Fig. 6c, show a clear T_2_ (negative) contrast enhancement as the ‘Fe’ concentration increases. It is seen that the MGO 3 produced an enhanced T_2_ contrast due to its higher relaxivity. Figure 6d shows a variation of r_2_ values of MGOs with D_H_.

**Figure 7:**
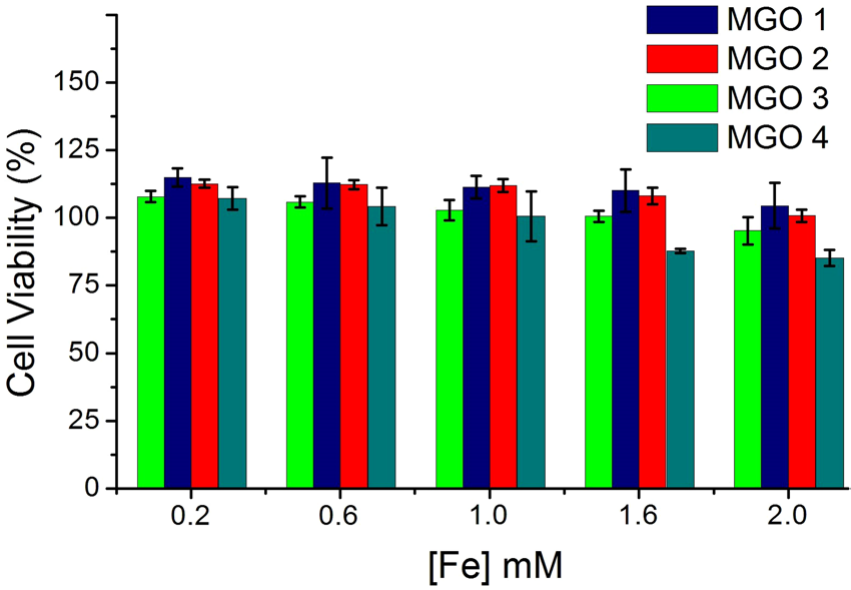
MTS assays of MGOs in MDA-MB-231 human breast adenocarcinoma epithelial cells.

Besides the evaluation of MR efficacy of MGOs, the study of toxic responses of such materials is crucial in MRI applications. We studied the cell viability of human breast adenocarcinoma epithelial cells (MDA-MB-231) when interacted with MGOs via 3-(4,5-dimethylthiazol-2-yl)-5-(3-carboxymethoxyphenyl)-2-(4-sulfophenyl)-2H-tetrazolium (MTS) assay. The results, in Fig. 7, show that the MGOs have high biocompatibility, in general, which is consonant with previous reports^23,24^. The fact that MGO 3 has the highest relaxivity and remains nontoxic at least up to 2 mM of [Fe] is very promising for contrast agent applications, notwithstanding the fact they have lower relaxivities than SPIOs^28,29^ and would require higher concentrations. As explained by Garg *et al*.^49^, nanosized GOs show very low toxicity and can be further improved by surface engineered biocompatible polymers, which may enable them to be effectively employed as contrast agents.

## Conclusion

Magnetic graphene oxide with different weight percentages of iron oxide nanoparticles decorated on graphene oxide were synthesized in nanocomposite form. The MGOs exhibit tunable magnetic behavior and hydrodynamic sizes consistent with the weight percentages of the constituents. They show distinct MR transverse relaxivity (r_2_) values of 27 ± 0.527, 106 ± 3.752, 162 ± 5.703 and 103 ± 8 mM^−1^s^−1^ corresponding to the hydrodynamic sizes of 376, 409, 414 and 434 nm respectively.

These diverse r_2_ values associated with the hydrodynamic sizes are analogous to those observed in the theoretical size regimes defined as motional averaging regime (MAR), static dephasing regime (SDR) and echo-limiting regime/slow motion regime (ELR/SMR) in classical outer sphere relaxation theory. Such behavior was previously observed only in the single core or cluster forms of IO. Further, the MGOs demonstrate excellent colloidal stability in aqueous solution and *in vitro* cytocompatibility in cancer cells. These show how to achieve the optimization of transverse relaxivity of magnetic graphene oxide and paves the way for the MRI applications of graphene-based nanocomposite magnetic materials.

## Methods

### Materials

Ferric chloride hexahydrate (FeCl_3_.6H_2_O, ≥99%), Ferrous chloride tetrahydrate (FeCl_2_.4H_2_O, 99.99%), Ammonium hydroxide (NH_4_OH, 28.0–30.0%), Sodium chloride ≥99.5%, Hydrochloric acid 37%, Sodium nitrate (NaNO_3_, ≥99.0%), Potassium permanganate (KMnO_4_, ≥99.0%) were purchased from Sigma-Aldrich.

### Synthesis of magnetic graphene oxide (MGO)

The MGO was synthesized in the form of IO-GO nanocomposite following the protocol reported previously^43^ with minor modification. Firstly, the spherical IO were synthesized via the co-precipitation method^36^. In general, 4.14 gm of FeCl_3_.6H_2_O and 1.62 gm of FeCl_2_.4H_2_O was dissolved in 75 mL of deionized water by mechanical stirring. The pH of the solution was adjusted to 10.57 by adding NH_4_OH solution under N_2_ atmosphere and vigorous stirring followed by 1 h aging at 70 °C. After cooling the solution, the product was washed via magnetic decantation for 5 times, and the powder form of IO was obtained after the lyophilization. Secondly, the GO was synthesized via the modified Hummer and Offeman’s method^50^. Following the washing process, the GO solution was probe sonicated for 24 h at 20% amplitude with 30 s ‘ON’ and 60 s ‘OFF’ to generate the GO flakes. For the synthesis of IO-GO nanocomposite, 240 mg of GO was gently sonicated to dissolve in 50 ml of deionized (DI) water (~5 mg/ml) followed by the addition of 20 mg of IO. The mixture was aged for 12 h (overnight) at room temperature with constant mechanical stirring to obtain the nanocomposite as MGO 1. Finally, the product was washed using magnetic decantation three times with DI water and was dried by lyophilization. The IO:GO at the ratio of 2:12, 3:12 and 4:12 (w/w) were used for the synthesis of MGO 2, MGO 3 and MGO 4, respectively.

### Characterization and cell viability assay

The thermogravimetry (TG) was performed using a PerkinElmer STA 6000 Simultaneous Thermal Analyzer by heating 7 mg of each MGO powder in the temperature range of 30 to 800 °C at the rate of 5 °C per minute in the presence of a constant N_2_ flow of 20 ml/min. The inductively coupled plasma optical emission spectrometry (ICP-OES) was carried out using Optima 8000 Perkin Elmer ICP–OES (PerkinElmer, Inc.). Samples were prepared according to the procedure previously reported^51,52^ with a minor modification (see Table S1 in SI). The energy dispersive X-ray (EDX) spectra were obtained in a scanning electron microscope (SEM) JEOL JSM-5800LV with operating voltage of 20 kV. The SEM and TEM images were obtained using a JOEL JSM 7500 F Field emission scanning electron microscope (FE-SEM) and a JEOL JEM 2100 F Field emission transmission electron microscope (FE-TEM) with operating voltage of 200 kV. The X-ray diffraction (XRD) patterns were obtained by a Rigaku SmartLab X-Ray diffractometer using CuK_α_ (λ = 1.5406 Å) operating at 40 KV and 44 mA. The attenuated total reflectance Fourier transform infrared (ATR-FTIR) spectra were obtained by a Bruker Tensor 27 spectrometer. The Raman spectra were obtained using a Thermo Scientific DXR Confocal Raman Microscope. The X-ray photoelectron spectroscopy (XPS) was performed on a Kratos Axis Ultra X-ray photoelectron spectrometer (Kratos Analytical, Inc., Manchester, UK) using monochromatic Al Kα radiation (hν = 1486.6 eV). High resolution spectra were collected using a 0.7 mm × 0.3 area. The powder samples were loaded for analysis onto double-sided copper tape. The dynamic light scattering (DLS) and Zeta potential were performed using a Malvern Zetasizer Nanoseries Nano-ZS (Malvern Instruments, Malvern, UK) operating a helium-neon laser wavelength of 633 nm and power of 4 mW. The magnetic measurements were performed in a physical property measurement system (PPMS) DynaCool (Quantum Design, Inc.). The magnetic resonance (MR) transverse relaxivity (r_2_) measurements were conducted on the NMReady-60PRO benchtop relaxometer (Nanalysis Corp. Canada) at 1.41T at 30 °C. The T_2_-weighted magnetic resonance (MR) phantom images were obtained by Agilent 4.7T preclinical MRI scanner at 4.7T. The cell viability studies on human breast adenocarcinoma epithelial cells, MDA-MB-231 were analyzed via MTS assay as described in detail previously by our group^36,53^.

### Ethics Approval

All cell experiments were performed in accordance with the protocols approved by the Biosafety Committee at the Molecular Sciences Research Center, University of Puerto Rico, San Juan, PR 00926, USA.

## Supplementary information

Supporting Information

## Data Availability

All data obtained and analyzed in this research work are included in this published article and its Supplementary Information file.
