# Supplementary material for: Controlling the transverse proton relaxivity of magnetic graphene oxide
Source: Sci Rep. 2019 Apr 4;9:5633. doi: 10.1038/s41598-019-42093-1 (PMC6449378; doi:10.1038/s41598-019-42093-1)
Supplement: Supplementary file 1 — Supporting Information [file 41598_2019_42093_MOESM1_ESM.pdf]

Supporting Information

Controlling the transverse proton relaxivity of magnetic graphene oxide

Bibek Thapa[1,2,*], Daysi Diaz-Diestra[1,3], Dayra Badillo-Diaz[1,4], Rohit Kumar Sharma[1,5], Kiran Dasari[1,6], Shalini Kumari[7], Mikel B. Holcomb[7], Juan Beltran-Huarac[1,8,*], Brad R. Weiner[1,3], and Gerardo Morell[1,2]

[1] Molecular Sciences Research Center, University of Puerto Rico, San Juan, PR 00926, USA

[2] Department of Physics, University of Puerto Rico, Río Piedras Campus, San Juan, PR 00925, USA

[3] Department of Chemistry, University of Puerto Rico, Río Piedras Campus, San Juan, PR 00925, USA

[4] Department of Biology, University of Puerto Rico, Río Piedras Campus, San Juan, PR 00925, USA

[5] Department of Environmental Sciences, University of Puerto Rico, Río Piedras Campus, San Juan, PR 00925, USA

[6] Department of Electrical and Computer Engineering, University of Illinois at Urbana-Champaign, Urbana, IL 61801, USA

[7] Department of Physics & Astronomy, West Virginia University, Morgantown, WV 26506, USA

[8] Center for Nanotechnology in Drug Delivery, UNC Eshelman School of Pharmacy, University of North Carolina, Chapel Hill, NC 27599, USA

- Corresponding author:

Email address: bibek.thapa@upr.edu, bibech.thapa@gmail.com (B.T)

and juan.beltran.huarac@gmail.com (J.B.H)

Sample name	Wt. of Sample (mg)	Wt. of ‘Fe_3O_4’ (mg)	Wt. of ‘Fe’ (mg)	Volume of 70% HNO_3 used to digest ‘Fe’ (ml)	Final volume with 2% HNO_3 (ml)	#Calculated conc. (ppm or mg/L)	*Ave. observed conc. from ICP-OES (ppm or mg/L)	*Ave. observed intensity	Error %
MGO 1	4.0	0.4	$289.52*10^{-3}$	1	35	8.27	7.5	6612296.13	10.26
MGO 2	2.2	0.44	$318.08*10^{-3}$	1	35	9.08	8.24	7197786.77	10.19
MGO 3	4.0	1.08	$781.76*10^{-3}$	1	35	22.34	23.68	19389114.33	5.65
MGO 4	4.0	1.28	$926.8*10^{-3}$	1	35	26.48	26.3	21455164.17	0.68
0 ppm (Stndrd.)							0	-23611.8	
2 ppm (Stndrd.)							2.5	$2.46858*10^6$	
5 ppm (Stndrd.)							5	$5.12608*10^6$	
10 ppm (Stndrd.)							10	$8.49947*10^6$	
25 ppm (Stndrd.)							25	$2.08653*10^7$	
50 ppm (Stndrd.)							50	$4.146*10^7$	
75 ppm (Stndrd.)							75	$6.08507*10^7$	
100 ppm (Stndrd.)							100	$7.81683*10^7$	

Table S1: The #calculated data from TGA and *observed data from ICP-OES

MGO was digested with 1 mL of 70% nitric acid (HNO_3) for 24 hours, and the sample was reconstituted with deionized water to 2% of HNO_3. Then, the solution was filtered twice using Whatman glass microfiber filter (GF/F grade) to eliminate carbon materials. The quantification of ‘Fe’ concentration was triplicated with the help of an external calibration curve.

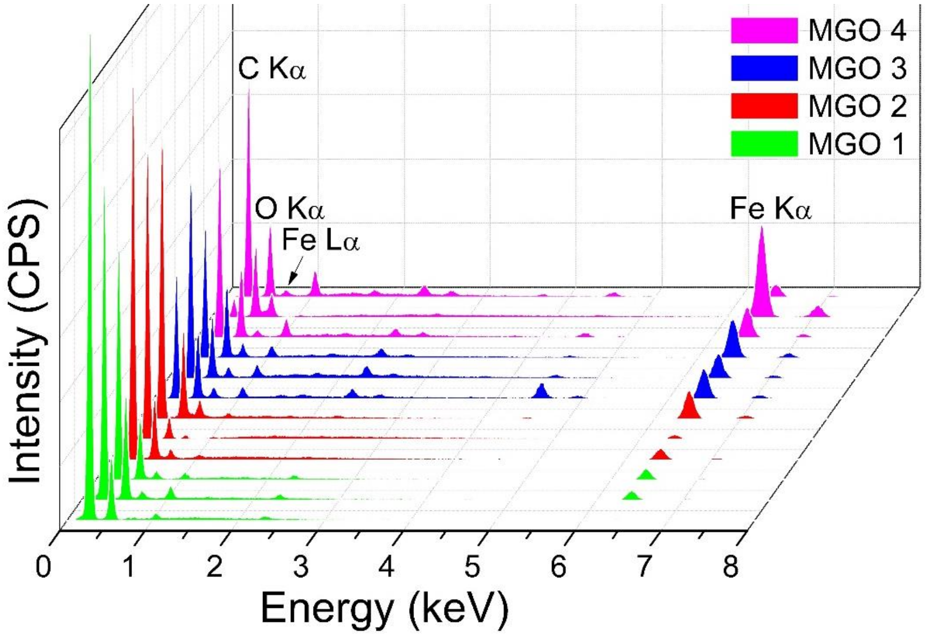

Figure S1: The energy-dispersive X-ray (EDX) spectra of MGOs

The elemental analysis of MGOs was carried out by EDX spectroscopy. The spectra were taken from three different regions in each MGO. As shown in Figure S2, the MGO 1, MGO 2, MGO 3 and MGO4 yield 7%, 12%, 20% and 25% ‘Fe’ weight in average. The spectra show different magnitude of intensity suggesting that the MGOs do not have uniform (homogeneous) IO distribution.

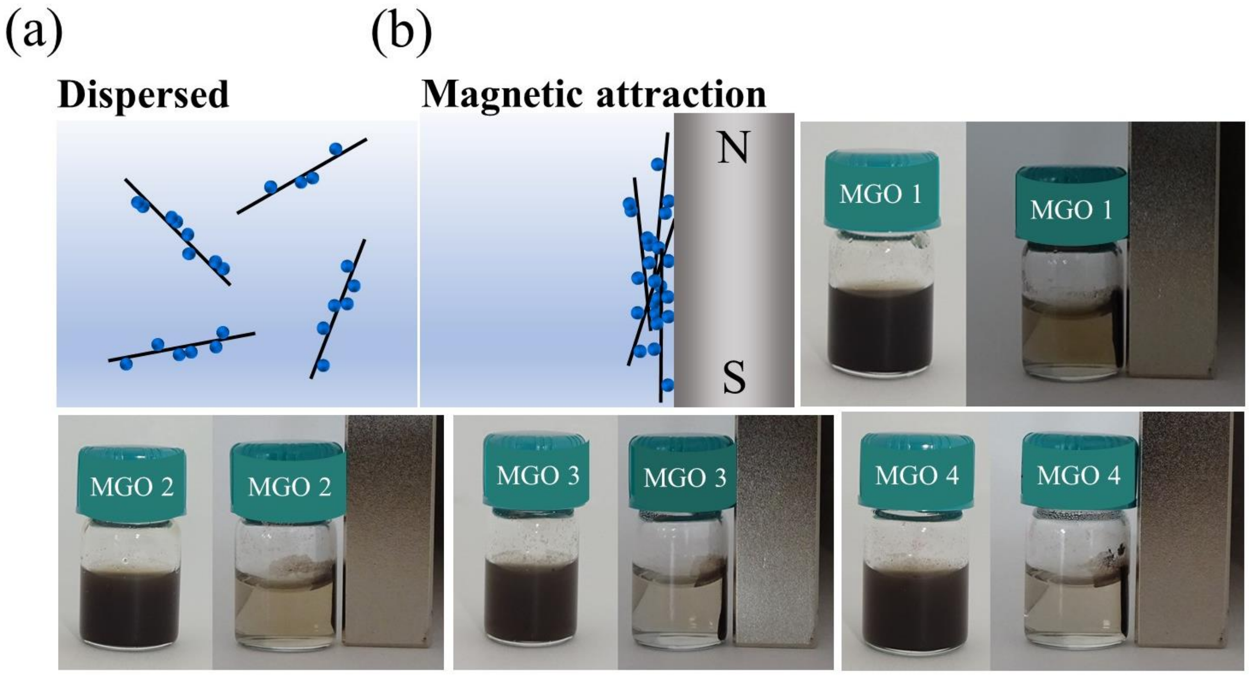

Figure S2: (a) Schematic representation of aqueous dispersibility and magnetic attraction of MGO.

(b) Optical images of aqueous dispersed and magnetically attracted MGOs.

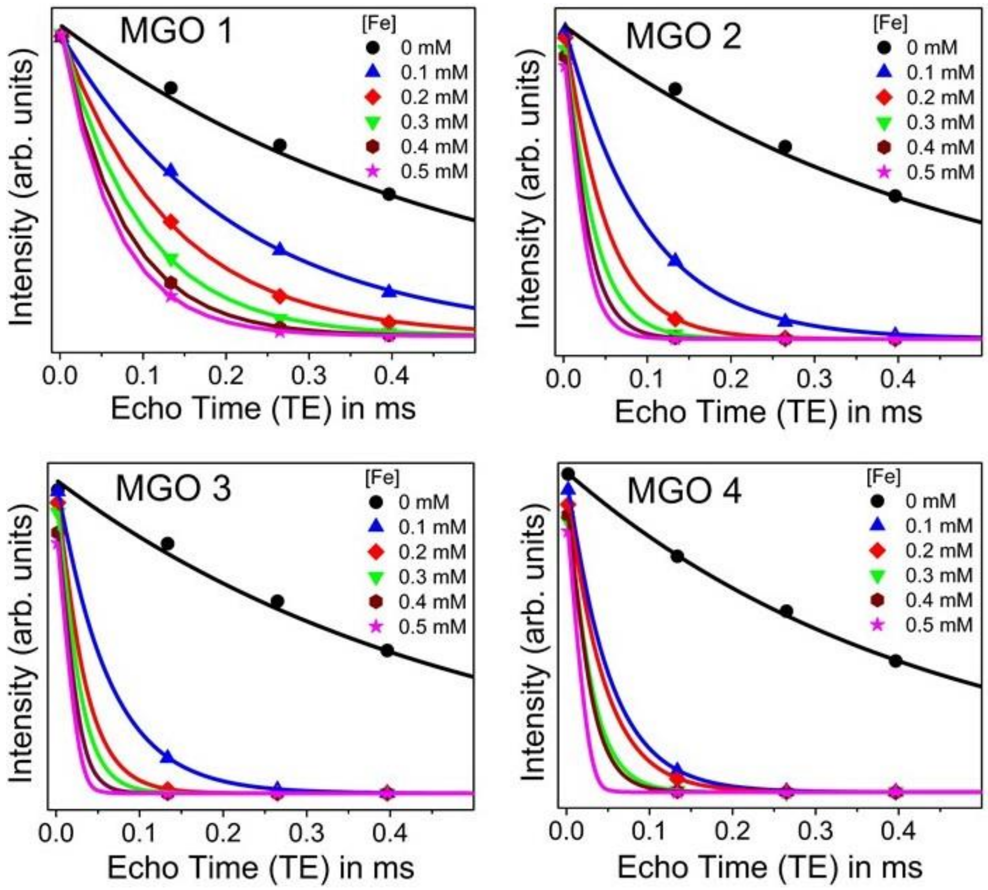

Figure S3: MR T_2 decay curves of MGOs with different [Fe] as indicated
